# GLUT3 as an Intersection of Glycerophospholipid Metabolism and the Innate Immune Response to *Candida albicans*

**DOI:** 10.3389/fcimb.2021.648988

**Published:** 2021-06-18

**Authors:** Xian Wu, Ge Zhang, Wen-Hang Yang, Jing-Tao Cui, Li Zhang, Meng Xiao, Ying-Chun Xu

**Affiliations:** ^1^ Department of Laboratory Medicine, State Key Laboratory of Complex Severe and Rare Diseases, Peking Union Medical College Hospital, Chinese Academy of Medical Science and Peking Union Medical College, Beijing, China; ^2^ Beijing Key Laboratory for Mechanisms Research and Precision Diagnosis of Invasive Fungal Diseases, Peking Union Medical College Hospital, Beijing, China; ^3^ Graduate School, Peking Union Medical College, Chinese Academy of Medical Sciences, Beijing, China

**Keywords:** GLUT3, Candida albicans, innate immune response, glycerophospholipid metabolism, immunometabolic

## Abstract

Immune cells can optimize the management of metabolic resources to balance their energy requirements in order to regulate immune responses. The interconnection between immunometabolism and fungal infections is becoming increasingly apparent. Using proteome and metabolome assays, we found that stimulation of primary human monocytes by *Candida albicans* was accompanied by upregulation of glucose transporter 3 (GLUT3) and activation of the glycerophospholipid metabolism pathway. Upregulated GLUT3 expression has been preliminarily confirmed in monocytes from patients with *C. albicans* bloodstream infection. Our findings support the importance of GLUT3 in the complex network of glycerophospholipid metabolism and the innate immune responses against *C. albicans*. In summary, this study might contribute to decipher the regulatory mechanism between the monocyte metabolic reprogramming and innate immune response and reveal potential targets for the antifungal treatments.

## Introduction

The human immune system is inextricably linked to metabolic systems. These two systems share common regulatory mechanisms that allow for coordination and extensive bidirectional communication (Odegaard and Chawla, 2013). Increased evidence supports the notion that metabolic reprogramming in immune cells is required for the response to microbial infections (Arts et al., 2016; Lachmandas et al., 2016; O’neill and Pearce, 2016; Domínguez-Andrés and Arts, 2017). *Candida albicans* is an opportunistic pathogen, which represents a severe threat to human life in immunocompromised individuals. Especially when *C. albicans* causes invasive fungal diseases, morbidity and mortality may exceed 50% even with antifungal therapy (Schultz et al., 2020).

The innate immune system is typically the first-line defense against *C. albicans* infection, and monocytes play a pivotal role in the process of antifungal immunity. (Qin et al., 2016; Grondman et al., 2019). Monocyte deficiency is associated with higher susceptibility to fungal infections in both mice and humans (Ngo et al., 2014; Ceesay et al., 2015; Domínguez-Andrés and Arts, 2017). When monocytes are stimulated and activated by pathogen-associated molecular patterns (PAMPs), glucose metabolism associated with ATP production is altered from aerobic oxidation to glycolysis (Palmer et al., 2016). While ensuring the energy supply for basic cellular processes, aerobic glycolysis fuels the production of nucleic acids and lipids required for the proliferation and differentiation of monocytes during the immune response (Raulien et al., 2017). However, not all the metabolic changes promote adequate immune responses. Previous studies have indicated that metabolic reprogramming after immune stimulation may be a major mechanism driving immune cell tolerance (Cheng et al., 2016; Kan and Michalski, 2018; Zhu et al., 2019). Moreover, the loss of metabolic plasticity affects the antifungal immune functions of monocytes (Grondman et al., 2019). Therefore, monocyte metabolic remodeling acts as a “double-edged sword” in regulation of the innate immune function.

In order to understand the role of cellular metabolism in the innate immune function of monocytes, human primary monocytes were stimulated with heat-killed *C. albicans* and proteomic and metabolomic analyses were performed. We found that stimulation of monocytes by *C. albicans* was accompanied by upregulation of glucose transporter 3 (GLUT3) and activation of the glycerophospholipid metabolism pathway. Upregulated GLUT3 expression has been preliminarily confirmed in monocytes from patients with *C. albicans* bloodstream infection (BSI) and GLUT3 knockdown by siRNA significantly reduced production of IL-1β in THP-1 cells. In addition, the glycerophospholipid metabolites could promote the localization and glucose uptake function of GLUT3 (Hresko et al., 2016). Our findings support the importance of GLUT3 in the complex network of glycerophospholipid metabolism and the innate immune responses against *C. albicans*.

## Materials and Methods

### Isolation of Primary Cells and CD14^+^ Monocytes

Peripheral blood mononuclear cells (PBMCs) were isolated from blood diluted in PBS (1:1) by differential density gradient centrifugation using Ficoll-Paque PLUS (GE Healthcare). Cells were washed twice in PBS (HyClone) and resuspended in RPMI 1640 culture medium (HyClone). Monocytes were isolated from the PBMCs using magnetic beads labeled with anti-CD14 without CD16 depletion (STEMCELL).

### Cell Culture and Treatment

*C. albicans* SC5314 (ATCC MYA-2876) was grown on Sabouraud dextrose agar plates to generate yeast cells at 29°C for 36 h. The cells were collected by centrifugation, washed twice with PBS, and heat-killed for 30 min at 95°C. Monocytes (5×10^6^ cells/mL) were stimulated with the heat-killed *C. albicans* yeast (MOI = 1) for 12 h and 24 h. Stimulated monocytes, including heat-killed yeast, were washed twice with PBS, harvested by centrifugation in sterile centrifuge tubes, snap frozen in liquid nitrogen, and shipped to perform proteomic and metabolomic tests (BIOMS).

### Protein Extraction, Proteomics Database Search, and Analysis

Liquid chromatography with tandem mass spectrometry (LC-MS/MS) analysis was conducted using an Orbitrap Fusion Lumos mass spectrometer (Thermo Fisher Scientific) combined with an EASY-nLC 1000 nanoflow system (Thermo Fisher Scientific). The MS data were analyzed by MaxQuant software (version 1.5.3.8). The false discovery rate (FDR) was set to 0.01 for both peptide and protein levels based on the q-value. The human Uniprot database (version 05/2019) with 73,939 protein entries was used for the proteomics search. Evaluation of regulatory events between different sample groups was achieved using two-sided Student’s *t*-tests, and the differentially expressed proteins were defined as *P* < 0.05, with a fold change ≥ 1.5, or ≤ 0.7. For differential proteins, principal component analysis (PCA) was performed through dimension reduction analysis of the data to detect the differences between the experimental groups and the repeatability within the groups. PCA was performed using Perseus software (version 1.5.5.1), and the diagram was generated using ATP-BioCloud analysis software (http://cloud.aptbiotech.com/#/). Venn diagrams and heat maps representing the normalized abundance of sample groups were drawn using TBTools software (https://github.com/CJ-Chen/TBtools/releases). The raw datasets are available in the ProteomeXchange Consortium (iPROX) repository under accession number PXD024515.

### Metabolites Extraction, Metabolomic Database Search, and Analysis

The samples were vortexed for 30 s. After three cycles of liquid nitrogen freezing and thawing, the samples were centrifuged at 12,000 rpm at 4°C for 20 min, and the supernatant was collected for freezing. Methanol was added for refluxing, and the samples were sonicated for 5 min. Finally, the samples were centrifuged at 12,000 rpm at 4°C for 20 min, the supernatant was collected and stored for LC-MS/MS analysis. A quality control (QC) sample was prepared and injected three times at the beginning of the run to ensure equilibration of the system. The QC sample was re-applied between every 10 samples during the run to ensure consistency of the analysis.

Sample analysis was performed on an ExionLC (SCIEX) coupled with a TripleTOF 5600+ (SCIEX) in both positive and negative ionization modes. The raw metabonomic data was imported to Progenesis QI (Waters) for peak alignment to obtain the retention time, molecular mass data (m/z), and peak area of each sample on the peak list. As the first step of EZinfo software analysis, PCA was mainly used to reduce the dimension of the data and to detect the difference between the experimental groups and the repeatability within the groups. Then PCA was performed by Progenesis QI software and the diagram was drawn by ATP-BioCloud analysis software. Differential metabolites were screened using the EZinfo software built-in statistical analysis method. Metabolites selected for further statistical analysis were identified on the basis of a coefficient of variation < 30%, variable importance in the projection (VIP) > 1, and *P* value < 0.05. The Human Metabolome Database (HMDB) (http://www.hmdb.ca), Kyoto Encyclopedia of Genes and Genomes (KEGG) database (http://www.genome.jp/kegg/), and National Institute of Standards and Technology (NIST) database (https://www.nist.gov/srd) were used to align the m/z values to identify metabolites. MetaboAnalyst (https://www.metaboanalyst.ca) was used to identify the metabolic pathways.

### Flow Cytometry

We collected samples from age-, sex-, and basic disease-matched populations, excluding *C. albicans* BSI patients and healthy donors with hyperthyroidism and a history of insulin injection, because these conditions may affect the expression of GLUT3. The demographic information and clinical characteristics of the subjects are listed in **Table 1**. PBMCs from patients and healthy controls were extracted as described above. Isolated cells were stained with PerCP-conjugated anti-CD3, PE-conjugated anti-CD14 (BioLegend) and FITC-conjugated anti-GLUT3 (R&D Systems) to detect the expression of GLUT3 in monocytes and analyze using FlowJo 10.0 software.

**Table 1:** Demographic and clinical characteristics of subjects.

Characteristic	Normal controls	*C. albicans* BSI patients
Subject no.	10	9
Age (years, mean ± SD)	48 ± 7	47 ± 13
Male (No, %)	6 (60.0)	6 (66.7)
Han Ethnic (No, %)	10 (100.0)	8 (88.9)
mono% (%, mean ± SD)	5.96 ± 1.61	5.74 ± 2.45^a^
mono# (10^9^/L, mean ± SD)	0.57 ± 0.31	0.45 ± 0.17^a^
FPG (mmol/L, mean ± SD)	5.36 ± 0.43	6.06 ± 1.65^a^
Hyperthyroidism (No, %)	0 (0.0)	0 (0.0)
History of insulin injection (No, %)	0 (0.0)	0 (0.0)

Compared with the normal controls group, ^a^P＞ 0.05. FPG, fasting plasma glucose.

### THP-1 Cell Culture, siRNA Delivery, and Cytokine Detection

THP-1 human acute monocyte leukemia cells were cultured in RPMI 1640 supplemented with 10% FBS (Gibco). GLUT3 siRNA or negative control (Invitrogen) was transfected to THP-1 cells for 24 h by HiPerFect Transfection Reagent (Qiagen) and incubated with heat-killed *C. albicans* yeast (MOI = 1) for a further 24 h. The cells were used for RNA extraction, and culture supernatants were collected for analysis of *in vitro* IL-1β, IL-6, and TNF-α production using the ELISA kit (Elabscience). The siRNA control was represented by a non-specific Stealth RNAi^®^ Negative Control.

### Statistical Analysis

GraphPad Prism 8.0 software was used to conduct statistical analyses, and the comparison between protein and metabolite levels was performed using the Student’s *t*-test. The non-parametric Mann-Whitney U test was used to determine the differences between *C. albicans* BSI patients and normal controls. Correlations between variables were evaluated using the Spearman rank correlation test. The comparisons between the mRNA level and IL-1β, IL-6, TNF-α secretion were performed using the paired *t*-test and all *P* values < 0.05 were considered statistically significant.

## Results

### Overview of the Multi-Omic Analysis of Monocytes Stimulated by *C. albicans*

**Figure 1:**
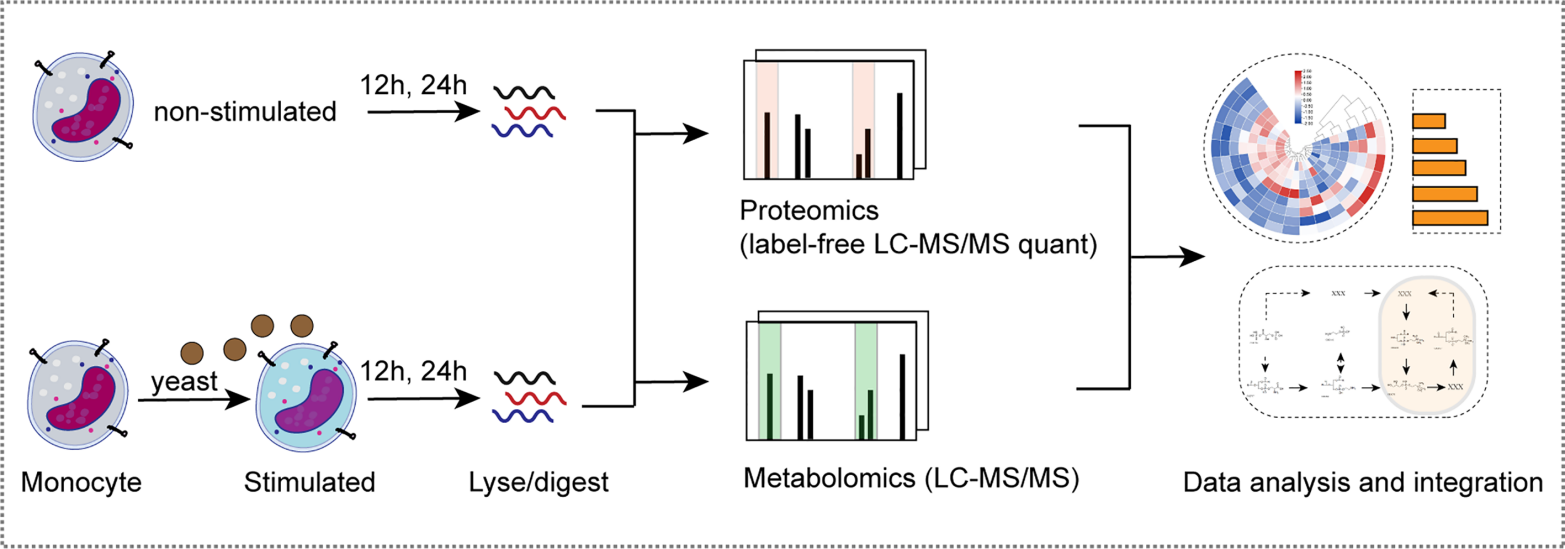
Multi-omics analysis of monocytes stimulated by *C. albicans*. Schematic view of the experimental investigation of monocytes stimulated by *C. albicans*.

Monocytes are innate immune cells that play a pivotal role in antifungal immunity. To better understand the mechanism of monocyte metabolic remodeling and immune regulation, we generated proteomic and metabolomic profiles for primary monocytes from healthy volunteers (n = 5) stimulated with *C. albicans* for 12 h (Y-12 h) and 24 h (Y-24 h). Proteins were digested and analyzed by label-free LC-MS/MS. In parallel, polar metabolites were extracted from monocytes at each time point and analyzed using non-targeted metabolomics. Furthermore, we employed a bioinformatics analytical approach to identify and analyze the immunometabolic pathways among the different experimental groups (**Figure 1**).

### Proteomic Analysis of Monocytes Stimulated by *C. albicans*

**Figure 2:**
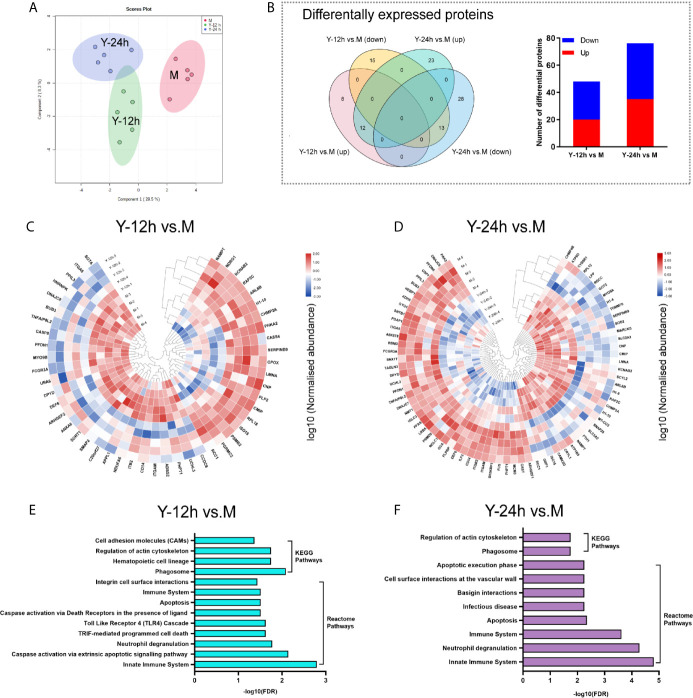
Landscape of proteome analyses. **(A)** Principal component analysis (PCA) of unstimulated monocytes (M) and monocytes stimulated with heat-killed *C. albicans* for 12 h (Y-12 h) and 24 h (Y-24 h) (n = 5). **(B)** Proteins differentially expressed in monocytes after 12 h and 24 h stimulation by heat-killed *C. albicans* versus M group were displayed in Venn diagrams. Bar graph shows the number of up- (red) and down-regulated (blue) proteins. **(C, D)** Hierarchical clustering analysis of differential proteins between the Y-12 h or Y-24 h and M group with a *P* < 0.05 (Student’s *t*-test). Normalized abundances of proteins were logarithmic transformed. **(E, F)** Pathway analysis of the differentially expressed proteins in monocytes stimulated by heat-killed *C. albicans*.

The PCA of the proteome data, as depicted in **Figure 2A**, clearly demonstrated the separation of the three different groups. As shown in the Venn diagram, after stimulation with yeast, comparisons of Y-12 h *versus* unstimulated monocytes (M) and Y-24 h *versus* M shared 25 differentially expressed proteins (12 upregulated and 13 downregulated), with 48 (20 upregulated and 28 downregulated) and 76 differentially expressed proteins (35 upregulated and 41 downregulated), respectively. The bar graph showed the upregulated and downregulated proteins in monocytes after 12 h and 24 h of stimulation (**Figure 2B**). Using hierarchical cluster analysis, the differentially expressed proteins for mapping the landscape of the Y-12 h or Y-24 h groups could be distinguished from the unstimulated group (**Figures 2C, D**). The signaling pathways in monocytes were further selected using the Search Tool for the Retrieval of Interacting Genes/Proteins (STRING) by filtering for differentially expressed proteins (n ≥ 2), and the FDR was < 0.05. Reactome Pathway and KEGG analyses showed that the differentially expressed proteins were specifically enriched in the innate immune system for both 12 h and 24 h *C. albicans*-stimulated monocytes (**Figures 2E, F**).

### Glycerophosphocholine Metabolism Pathway Was Active in Monocytes Stimulated by *C. albicans*

**Figure 3:**
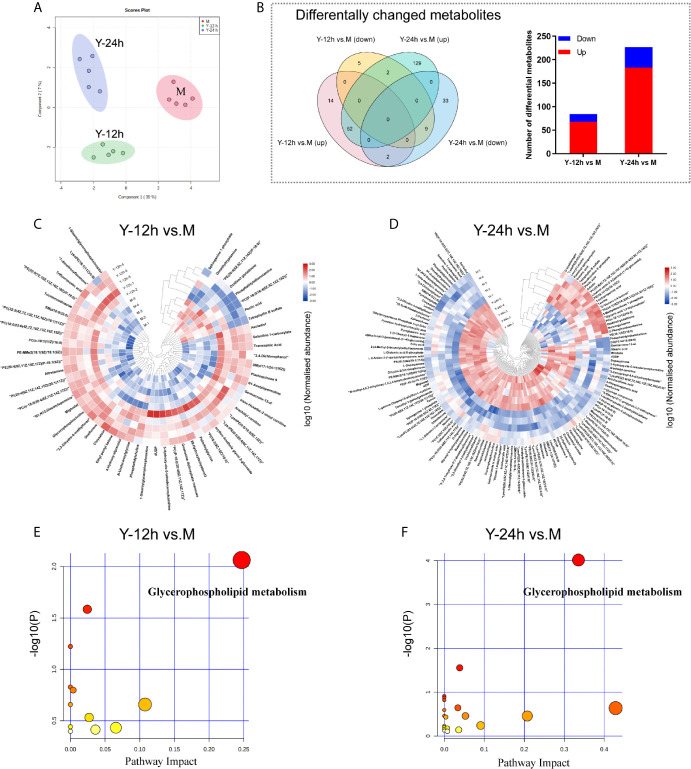
Data-dependent metabonomic analyses. **(A)** PCA of unstimulated monocytes (M) and monocytes stimulated with heat-killed *C. albicans* for 12 h (Y-12 h) and 24 h (Y-24 h) (n = 5). **(B)** Venn diagrams depict the differential metabolites of monocytes after 12 h and 24 h stimulation by heat-killed *C. albicans versus* M group. Bar graphs show the number of up- (red) and down-regulated (blue) metabolites. **(C, D)** Hierarchical clustering analysis of differential metabolites between monocytes stimulated by heat-killed *C. albicans* and the M group. Normalized abundance of metabolites was logarithmic transformed. **(E, F)** Pathway analysis of the differential metabolites in monocytes stimulated by *C. albicans*. The following criteria were used to identify relevant pathways: y axis, enrichment significance (*P* < 0.05, [log (*P*) > 1.3]) and x axis, pathway impact (> 0.2) for network topology.

To explore the metabolic changes in monocytes after stimulation by *C. albicans*, we used the same treatment protocol as that of the proteomics analyses to detect and analyze metabolomics. As shown in **Figure 3A**, a clear distinction between the M group and monocytes stimulated with *C. albicans* for 12 h and 24 h was observed in the PCA diagram, which was generated based on the metabolome data. After stimulation with *C. albicans*, the Y-12 h *versus* M and Y-24 h *versus* M comparisons shared 65 changed metabolites, including 52 upregulated and 11 downregulated, and the overlap between the two time points contained two metabolites that shifted from upregulated at 12 h to downregulated at 24 h. Comparisons of Y-12 h *versus* M and Y-24 h *versus* M individually showed 84 (68 upregulated and 16 downregulated) and 227 (183 upregulated and 44 downregulated) changed metabolites, respectively (**Figure 3B**, Venn diagram). The bar graph showed the upregulated and downregulated metabolites in monocytes after 12 h and 24 h of stimulation (**Figure 3B**). Using hierarchical cluster analysis of the changed metabolites to map a landscape, Y-12 h or Y-24 h monocytes could be clearly distinguished from the M group (**Figures 3C, D**). The differential metabolites were mainly enriched in the glycerophospholipid metabolic pathway (**Figures 3E, F**).

### GLUT3 and the Innate Immune Response in Monocytes

**Figure 4:**
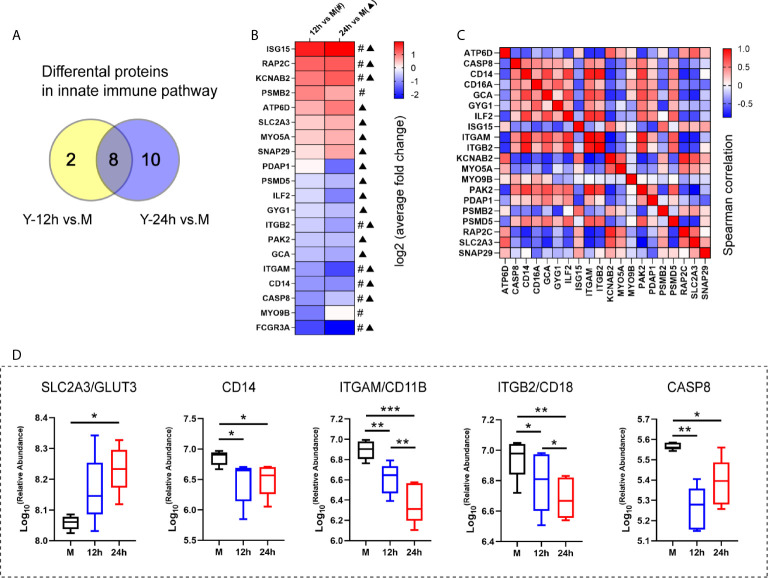
The analysis of the innate immune pathway-related proteins. **(A)** Venn diagrams depict the differential proteins in innate immune pathway after 12 h and 24 h stimulation by heat-killed *C. albicans versus* the M group. **(B)** The average fold changes of 20 differential proteins for those significantly regulated in at least one comparison group (Y-12 h *versus* M: # or Y-24 h *versus* M: ▲) were analyzed. The fold changes of proteins were logarithmic transformed. **(C)** Correlation matrix of innate immune pathway-related proteins. **(D)** Selected the up-regulated GLUT3 and down-regulated (ITGAM/CD11B, ITGB2/CD18, CD14 and CASP8) in the innate immune pathway are shown. ***P < 0.001; **P < 0.01; *P < 0.05.

Based on the results of the proteomics and metabolomics analyses, the relationship between the innate immune response and glycerophospholipid metabolism requires further exploration. The Y-12 h *versus* M and Y-24 h *versus* M groups shared 8 innate immune pathway-related proteins and individually had 10 and 18 differentially expressed proteins, respectively (**Figure 4A**). The heatmap shows the average fold changes of 20 differentially expressed proteins enriched in the innate immune response pathway. Compared to the M group, 8 differentially expressed proteins showed an increasing trend, while 12 differential proteins decreased (**Figure 4B**). Correlations between significantly regulated proteins in the innate immune pathway are shown in **Figure 4C**. Regarding the innate immune response pathway, the five proteins that we focused on are listed in **Figure 4D**, including four (namely CD14, ITGAM/CD11B, ITGB2/CD18, and CASP8) that were decreased, while GLUT3 was increased.

**Figure 5:**
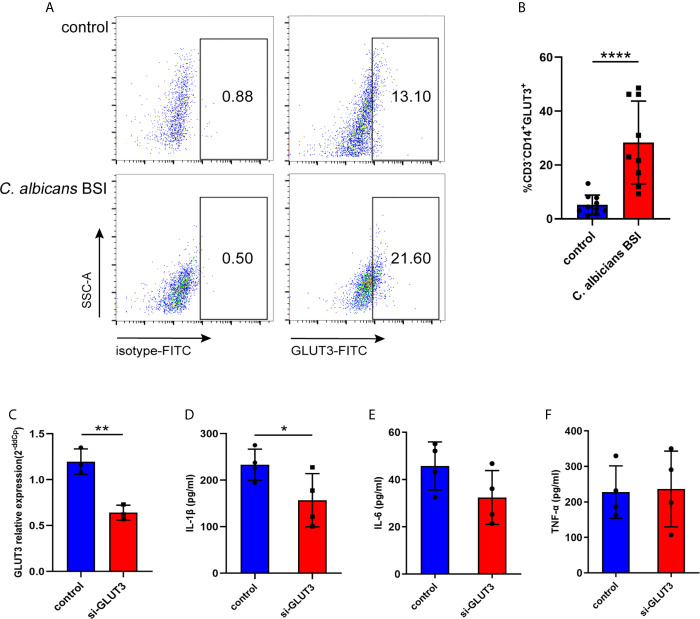
The GLUT3-expression in patients with *C. albicans* BSI was preliminarily confirmed in monocytes. PBMCs were isolated from patients (n = 9) and healthy controls (n = 10), and then CD3^-^CD14^+^GLUT3^+^ monocytes were separated by flow cytometer and compared using the non-parametric Mann-Whitney test (*P* < 0.0001). Representative flow cytometry dot plot **(A)** and summary data are shown **(B)**. GLUT3 knockdown by siRNA significantly reduced production of IL-1β in the THP-1 cells. **(C)** The GLUT3 mRNA expression achieved by transfection of si-GLUT3 or negative control to THP-1 cells (n = 3). **(D–F)** Comparison of the secretion of IL-1β, IL-6, and TNF-α in THP-1 cells stimulated by *C. albicans* for 24 h (n = 4). ****P < 0.0001; **P < 0.01; *P < 0.05.

To further characterize GLUT3 expression in the monocytes of patients with *C. albicans* BSI, CD3-CD14+GLUT3+ monocytes from patients (n = 9) and healthy donors (n = 10) were measured by flow cytometry. It was found that, compared to the healthy donors, the monocytes from patients showed upregulated expression of GLUT3 on the surface of the cell membrane (*P* < 0.0001; **Figures 5A, B**). To explore the effect of GLUT3 on the immune response of monocytes, siRNA-GLUT3 or negative control was delivered to THP-1 cells and the secretion of IL-1β, IL-6, and TNF-α was detected (**Figures 5C–F**). After successful knockdown of GLUT3 (*P* = 0.0035, **Figure 5C**), the si-GLUT3 and control THP-1 cells were treated with heat-killed *C. albicans* for 24 h. We found that the secretion of IL-1β in the supernatant decreased in si-GLUT3 group (*P* = 0.0364, **Figure 5D**), proving that expression of GLUT3 reductions would reduce the secretion of IL-1β.

### GLUT3 and the Glycerophospholipid Metabolism in Monocytes

**Figure 6:**
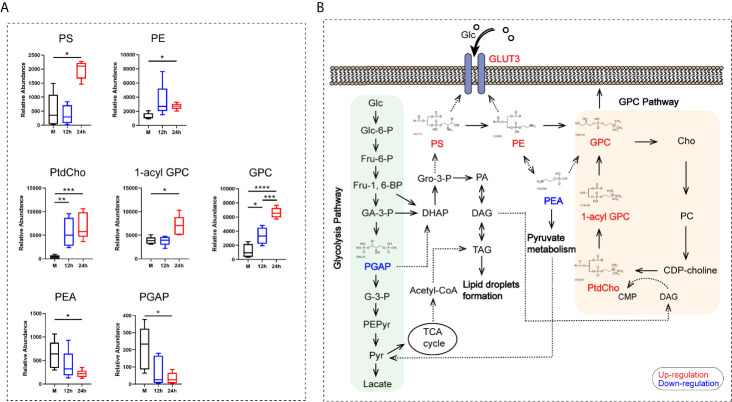
The analysis of the glycerophospholipid metabolism pathway-related metabolites. **(A)** Relative abundance of seven differential metabolites contributing to the GPC pathway of glycerophospholipid metabolism. **(B)** Schematic pathway map of the metabolites in the main metabolic pathways in monocytes stimulated with *C. albicans*. Glc, glucose; Glc-6-P, glucose-6-phosphate; Fru-6-P, fructose-6-phosphate; Fru-1,6-BP, fructose-1,6-bisphosphate; GA-3-P, Glyceraldehyde-3-phosphate; PGAP, glyceric acid 1, 3-biphosphate; G-3-P, 3-phosphoglycerate; PEPyr, phosphoenol- pyruvate; Pyr, pyruvate; DHAP, dihydroxyacetone phosphate; Gro-3-P, glycerol-3- phosphate; PA, phosphatidate; DAG, diacylglycerol; TAG, triacylglyceride; TCA, tricarboxylic acid cycle; PS, phosphatidylserine; PE, phosphatidylethanolamine; PEA, O-phospho-ethanolamine; GPC, glycerophosphocholine; Cho, choline; PC, phosphorcholine; CDP-choline, cytidine diphosphate choline; CMP, cytidine monophosphate; PtdCho, phosphatidylcholine; 1-acyl-GPC, 1-acyl-sn-glycero-3- phosphocholine; For all tests, significance values are denoted as follows: ****P < 0.0001; ***P < 0.001; **P < 0.01; *P < 0.05.

Metabolomic analyses showed that seven metabolites contributing to glycerophospholipid metabolism, namely phosphatidylserine (PS), phosphatidylethanolamine (PE), phosphatidylcholine (PtdCho), 1-acyl-sn-glycero-3- phosphocholine (1-acyl-GPC), glycerophosphocholine (GPC), O-phospho-ethanolamine (PEA), and glycericacid-1,3-biphosphate (PGAP), were mainly enriched after 24 h. Among these metabolites, PtdCho and GPC began to increase at 12 h after stimulation (**Figure 6A**). Our results revealed that the GPC pathway was activated in monocytes stimulated by heat-inactivated *C. albicans*, and all the metabolites listed above were directly or indirectly involved in the GPC pathway. We found a significant upregulation of the main metabolites involved in the GPC pathway, such as PtdCho, 1-acyl-GPC, and GPC. We also found that increased production of PS *via* PE was converted into GPC, followed by the conversion of GPC into Cho and transformation into PC and PtdCho (**Figure 6B**). Furthermore, previous studies have shown that PE promotes GLUT3 activation in monocytes, and PS is essential for GLUT3 function by activating and stabilizing its structure (Hresko et al., 2016). Consequently, these results support the idea that activation of the glycerophospholipid metabolism pathway and related metabolites could promote the function of GLUT3 during the innate immune response in monocytes stimulated with *C. albicans*.

## Discussion

*C. albicans* is the most common opportunistic fungal pathogen that causes a variety of human diseases, ranging from potential oral infections to life-threatening candidemia (Hasim et al., 2018). The development of immunotherapeutic approaches to boost host defense is urgently needed to reduce the mortality of *C. albicans* BSI (Pfaller and Diekema, 2007). Although increased evidence supports the interconnection between immunometabolism and fungal infections, we are just beginning to understand the metabolic reprogramming associated with the monocyte immune response and its impact on host defense against fungal infections (Tucey et al., 2018; Weerasinghe and Traven, 2020). Here, we describe the changes in proteins and metabolites in human monocytes after stimulation with heat-inactivated *C. albicans*. Briefly, we observed that monocytes stimulated with *C. albicans* for 24 h exhibited more significant changes in protein expression and metabolic activity than those stimulated for 12 h. Among these differentially expressed proteins in monocytes, the upregulation of GLUT3 may promote glucose transport and provide the energy needed for the innate immune response. Meanwhile, the structural stability and biological function of GLUT3 requires the participation of various glycerophospholipid metabolites, and concomitant with the activation of glycerophospholipid metabolism. Therefore, we speculate that GLUT3 is a critical intersection that participates in the innate immune response and glycerophospholipid metabolism.

Cellular glucose uptake is mediated by a family of GLUTs, which participate in immune and inflammatory responses and are critical for host defense against pathogens (Lang et al., 2020). GLUT3 is expressed and translocated to the cell membrane in response to a substantial increase in the energy demand associated with cell activation (Fu et al., 2004; Humphrey et al., 2004; Jones et al., 2006; Simpson et al., 2008). During infection, increased GLUT3 expression may help to redistribute glucose as a potential source of energy away from peripheral tissues and toward cells that mediate the immune response, and it is also essential for cell survival (Maratou et al., 2007). In addition, the differentiation of THP-1 monocytes into macrophages was associated with a significant induction of GLUT3 (Fu et al., 2004). GLUT3 is essential for monocytes to absorb energy for immune response function. However, few studies have focused on the relationship between GLUT3 expression and metabolic transformation in monocytes, especially glycerophospholipid metabolism.

Monocytes change their metabolic phenotype from oxidative to glycolytic metabolism when activated in humans (Palmer et al., 2016). However, based on our findings, there was no obvious activation of the pathways associated with glucose metabolism. Instead, the glycerophospholipid metabolism pathway, particularly the GPC pathway, was activated. Here, we briefly describe the basic features of these metabolites in the GPC pathway (**Figure 6B**). PtdCho is the most abundant phospholipid in mammalian cell membranes (Mountford and Wright, 1988; Sonkar et al., 2019). The first step in the GPC catabolic pathway is the hydrolysis of PtdCho to remove one fatty acid and produce 1-acyl GPC, which is subsequently converted to GPC by removing the second fatty acid, which is then converted to free choline (Cho) (Morash et al., 1988). In the synthesis pathway, free Cho is phosphorylated to phosphocholine (PC) and then converted to cytidinediphosphate (CDP)-choline to produce PtdCho, thus completing the GPC cycle (Sonkar et al., 2019).

The GPC metabolic pathway interacts with multiple biochemical pathways (Sonkar et al., 2019). PGAP is not only involved in glycerophosphate metabolism, but also is an important metabolite in glycolysis. Decreased expression of PGAP indicates that glycerol-3-phosphate (GA-3-P) tends to produce glycerol-3-phosphate (Gro-3-P) *via* dihydroxyacetone phosphate (DHAP), which promotes the synthesis of PS, PE, and GPC. As a product of fructose-1,6-bisphosphate (Fru-1,6-BP) in glycolysis, Gro-3-P is converted to phosphatidate (PA), which reversibly gets converted to diacylglycerol (DAG) or triacylglyceride (TAG), and then promotes the formation of lipid droplets. The DAG formed in this pathway was subsequently incorporated into the *de novo* synthesis of PtdCho (Sonkar et al., 2019). In addition, PEA participates in the pyruvate metabolism pathway and in the synthesis of PE and GPC. However, PEA and PGAP are indirectly involved in the synthesis of PS and PE, respectively, resulting in a decrease in their abundance (**Figure 6B**).

Previous studies have demonstrated that PE promoted GLUT3 activation in monocytes, and PS was found to be essential for GLUT3 function by activating and stabilizing its structure (Hresko et al., 2016). In this study, we found that PE and PS increased in monocytes after stimulation by heat-inactivated *C. albicans*, and both PE and PS were involved in the synthesis of GPC, promoting GPC cycling. These results suggest that PE and PS participate in the activation of GLUT3 *via* the GPC pathway, which contributes to the expression of GLUT3, localized at the cell membrane, thus facilitating immune response function. This prompted us to investigate the role of GLUT3 in the antifungal effector functions of monocytes against *C. albicians* infection. We found that downregulated GLUT3 significantly reduced the secretion of IL-1β in THP-1 cells stimulated with *C. albicans*. Pro-inflammatory cytokines IL-1β have been identified as integral components of human monocytes anti-fungal immune defenses (Ganesan et al., 2014; Gaidt et al., 2016).

The metabolic changes that we observed may correlate with the innate immune response after *C. albicans* stimulation. The downregulation proteins, such as ITGAM/CD11B, ITGB2/CD18, and CD14, involved in the innate immune pathway of monocytes, participate in the Toll-like receptor 4 (TLR4) signaling pathway. TLR4 is one of the receptors involved in the recognition of *C. albicans* and promotes the innate immune response (Ferwerda et al., 2008). TLR4 has been shown to be involved in proinflammatory responses in monocytes, macrophages, and dendritic cells, specifically, these immune cells combat *C. albicans* and require the release of cytokines that tend to rely on TLR4 pathway (Blasi et al., 2005; Qin et al., 2016; Wang et al., 2019). CD11B/CD18 (Mac-1/Complement Receptor 3, CR3) is an integrin molecule that is expressed on the surface of monocytes and plays an important role in their trafficking ability (Panni and Herndon, 2019). CD11B can modulate a variety of biological processes in innate immune cells, including the generation of type I interferon and proinflammatory cytokines (Khan et al., 2018). Notably, CD11b has also been implicated in the recognition of fungal β-glucan, which is a key PAMP located at the surface of *C. albicans* (Ballou et al., 2016). However, the downregulation of the inflammatory proteases caspase-8 (CASP8) levels may contribute to a reduction in the receptor-mediated apoptosis process (Mandal et al., 2020). A strong requirement for CASP8 and CR3 was also found for NLRP3 dependent IL-1β production in response to heat killed *C. albicans* (Ganesan et al., 2014).

Among the upregulated proteins that function in the innate immune response (**Figure S1** in the **Supplementary Materials**), ISG15 is known as an immunomodulator that controls fungal infection and triggers the expression of proinflammatory cytokines (Dong et al., 2017; Liu and Lee, 2021). RAP2C is a member of the Ras GTPase superfamily, which acts as a molecular switch to regulate cellular movement, proliferation, differentiation, and apoptosis (Wang et al., 2020; Zhu et al., 2020). KCNAB2 is a voltage-gated potassium channel subunit beta-2 that promotes expression of the pore-forming alpha subunits at the cell membrane and plays an important role in the regulation of membrane potential, therefore, it is important for the absorption of glucose, amino acids, and long-chain fatty acids (Mcdaniel et al., 2001; Wongdee et al., 2009). Both KCNAB2 and GLUT3 are membrane channel proteins that are important for glucose absorption. These results indicate that elevated levels of ISG15, RAP2C, and KCNAB2 are beneficial to the immune response to resist *C. albicans* infection, however, whether these proteins are related to the metabolites of glycerophospholipids requires further study.

Of note, the GPC pathway was activated gradually over 24 h. Previous research based on transcriptomic analysis has confirmed that the expression levels of several main enzymes involved in the glycolysis pathways were significantly upregulated, but this enhancement occurred after stimulation with heat-inactivated *C. albicans* for 24 h (Domínguez-Andrés and Arts, 2017). In our study, the protein abundance of GLUT3 also increased significantly after 24 h of stimulation. Monocyte activation and immune responses are processes that require high energy consumption and the metabolic transformation of monocytes needs to go through an interim stage (Palmer et al., 2016). To allow cells to adapt to these changes, an energy deficiency period is necessary to reduce energy consumption and ensure cell survival, resulting in dampening of the innate immune response. The expression and localization of GLUT3 could provide a channel for increased glucose uptake by monocytes and mobilize the metabolism of glycerophospholipids as a part of the signal transduction pathway.

### Research Limitations

In this study, we used heat-killed *C. albicans* to avoid the growth of live *C. albicans* that would consume the monocyte growth medium and lead to erroneous results, thereby reducing interference and clarifying the metabolic state of monocytes. However, heat-killed *C. albicans* did not elicit the same response as the metabolically active fungal cells. Thus, our results may not reflect what occurs *in vivo*. Therefore, the key proteins of the innate immune response and metabolites in the glycerophospholipid pathway need to be verified in the early stage of monocyte stimulation by *C. albicans in vivo* (infection model of mice). The mechanism of metabolism and the innate immune response of monocytes stimulated by *C. albicans* requires further validation.

## Conclusions

There is a complex regulatory network between the innate immune system and cell metabolism. Loss of cellular energy homeostasis may occur during the process of monocyte metabolic remodeling, eventually accounting for reduced antifungal immune response. Our findings suggest that GLUT3, as an intersection of glycerophospholipid metabolism and innate immunity, may promote energy absorption and restore the functional immune response. Clarifying the immunometabolic mechanism of the monocyte response to *C. albicans* infections is of great significance for exploring novel potential therapeutic targets.

## Data Availability Statement

The data presented in the study is deposited in ProteomeXchange, accession number: PXD024515.

## Ethics Statement

The studies involving human participants were reviewed and approved by Ethics Committee of Peking Union Medical College Hospital, Chinese Academy of Medical Sciences (JS-2782). Written informed consent for participation was not required for this study in accordance with the national legislation and the institutional requirements.

## Author Contributions

XW conceived and designed the experiment. GZ and W-HY contributed reagents/materials/analysis tools. XW and J-TC performed the experiments. XW analyzed the data and wrote the manuscript. Y-CX, MX, and LZ revised the manuscript. All authors contributed to the article and approved the submitted version.

## Funding

This work was supported by the National Natural Science Foundation of China (Grant No. 81971979, 81802049) and Beijing Key Clinical Specialty for Laboratory Medicine-Excellent Project (No. ZK201000).

## Conflict of Interest

The authors declare that the research was conducted in the absence of any commercial or financial relationships that could be construed as a potential conflict of interest.
