# Supplementary figures and images for: GLUT3 as an Intersection of Glycerophospholipid Metabolism and the Innate Immune Response to Candida albicans
Source: Front Cell Infect Microbiol. 2021 Jun 18;11:648988. doi: 10.3389/fcimb.2021.648988 (PMC8253260; doi:10.3389/fcimb.2021.648988)

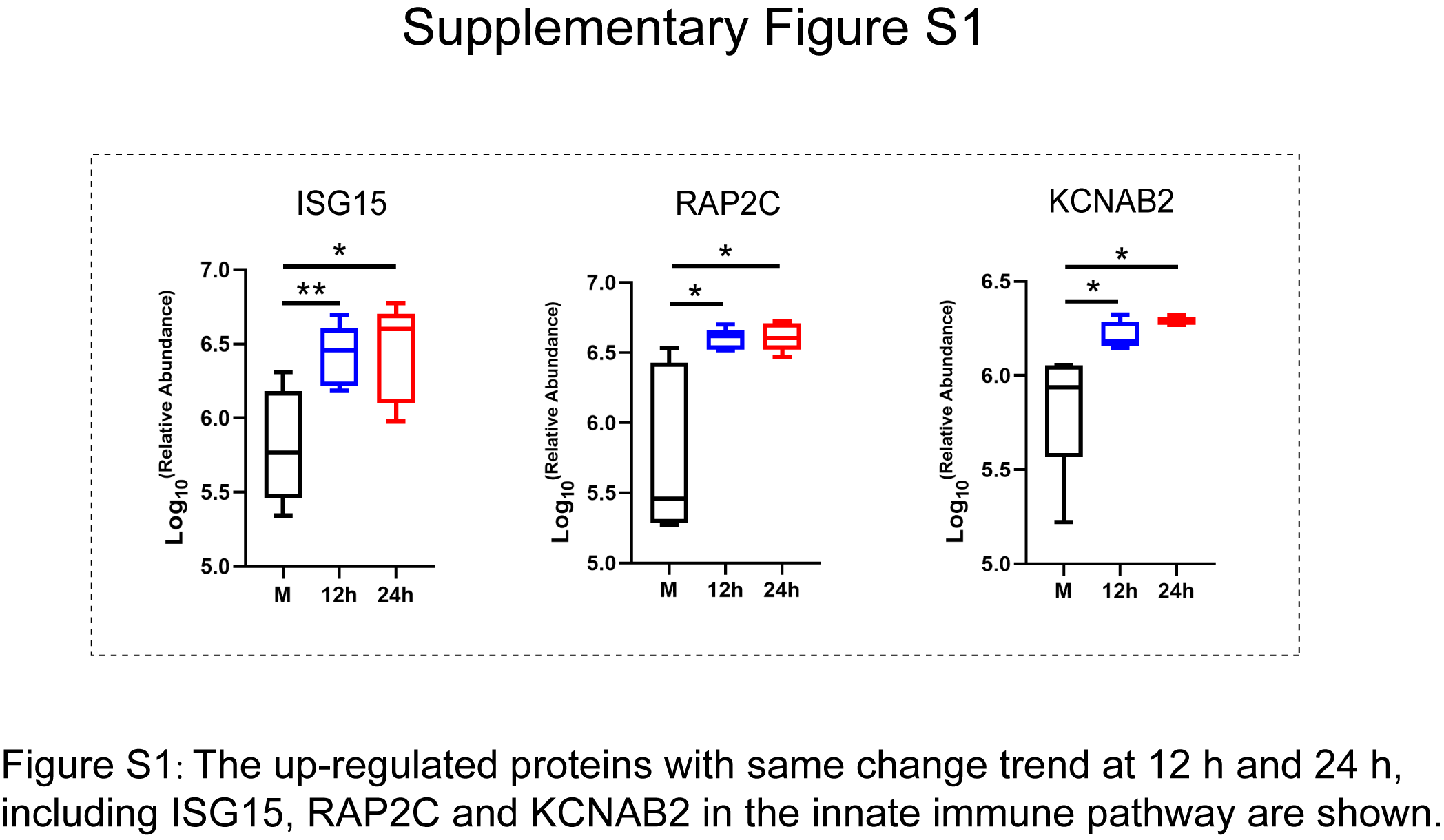

Supplement: Supplementary file 1 [file Image_1.tif]
